# Introduction to the Special Issue: *Prevention Science and Youth Conduct Problems: Development, Prevention, and Treatment*

**DOI:** 10.1007/s11121-025-01808-9

**Published:** 2025-04-21

**Authors:** Sarah J. Racz, Natalie Goulter, Yao Zheng

**Affiliations:** 1 Department of Psychology, University of Maryland at College Park, Biology-Psychology Building, College Park, MD 20742 USA; 2 College of Education, Psychology and Social Work, Flinders Institute for Mental Health and Wellbeing, Flinders University, Adelaide, SA Australia; 3 Department of Psychology, University of Alberta, Edmonton, AB Canada

**Keywords:** Conduct problems, Risk and protective factors, Prevention, Intervention, Treatment

## Abstract

This introductory article describes this Special Issue entitled *Prevention Science and Youth Conduct Problems: Development, Prevention, and Treatment* that is being offered in recognition of the research and scientific contributions of Dr. Robert J. McMahon. This Special Issue includes a collection of 15 original empirical research articles, systematic reviews, meta-analyses, and theoretical pieces spanning three themes consistent with Dr. McMahon’s program of research: (1) risk and protective factors in the development and maintenance of youth conduct problems; (2) family based preventive and treatment interventions for youth conduct problems; and (3) multicomponent preventive and treatment interventions for youth conduct problems. Following these articles, this Special Issue contains two commentaries from experts in the fields of youth conduct problems and prevention science, as well as a reflection from Dr. McMahon. Our introduction provides a brief synopsis of each article contained in the Special Issue, identifying how these works reflect upon and were inspired by Dr. McMahon’s research legacy and how they advance understanding of conduct problems. We close this introduction with thoughts regarding future research directions that will extend Dr. McMahon’s impressive impact on the field of youth conduct problems.

Decades of research on the development and maintenance of youth conduct problems have documented a multitude of associated risk and protective factors (e.g., genetics, temperament, prenatal development, parental and family characteristics, peer relationships, and community exposures) that interact with each other in dynamic ways (Kimonis et al., 2014; McMahon & Frick, 2005). What has also been established is the need for comprehensive and personalized interventions in order to see clinically significant improvements in youth conduct problems as they manifest across development (Frick, 2012; McMahon & Kotler, 2004). Three key avenues have been highlighted to enhance the effectiveness of preventive interventions targeting youth conduct problems: (1) identifying the most salient risk and protective factors that must be addressed in prevention and treatment; (2) incorporating the family system in prevention and treatment efforts, targeting parenting skills and the parent–youth relationship; and (3) implementing multicomponent prevention and treatment programs to address all relevant areas associated with youth conduct problems.

This Special Issue is being offered in honor of Dr. Robert J. McMahon’s research and scholarly contributions. After six decades as a scholar and contributor to the field of prevention science, he retired in September 2023 from his positions as Professor within the Department of Psychology at Simon Fraser University, BC Leadership Chair in Proactive Approaches to Reducing Risk for Violence among Children and Youth, and Director of the Institute for the Reduction of Youth Violence. Dr. McMahon also served as the Editor-in-Chief of *Prevention Science* from 2007 to 2013, building and maintaining a strong focus on quality scholarship at the intersection of prevention science and developmental psychopathology. Throughout his career, Dr. McMahon’s significant scientific contributions and impacts have focused largely on the three key areas identified above. His work encompasses a “bench to bedside” approach by advancing the understanding of the many risk and protective factors for youth conduct problems and directly translating that knowledge into actionable prevention and treatment approaches that target those problem behaviors. We know much more about how to intervene with youth conduct problems, and how to do so efficaciously and effectively, because of Dr. McMahon’s work.

In addition to his research productivity, Dr. McMahon has significantly influenced several generations of scholars who have furthered the science on the development, prevention, and treatment of child and adolescent conduct problems. Whether as a formal mentor to his students and supervisees, or as an informal mentor to early career researchers and other colleagues, his meticulous and detail-oriented work has inspired others to drive the field toward a deeper understanding of developmental psychopathology more broadly, and youth conduct problems in particular. The impact of his theory-driven empirical work, as well as the wide mentorship he has provided to so many, is reflected in the breadth and depth of the articles included in this Special Issue.

## Content and Organization of the Special Issue

Prior to Dr. McMahon’s retirement, we issued an open call for original articles to appear in this Special Issue of *Prevention Science*. We specifically chose this journal as an outlet for this Special Issue to recognize and honor Dr. McMahon’s leadership at the helm of *Prevention Science*—it seemed only fitting that papers that were inspired by and reflected upon his many scientific contributions appear in the journal that he led for so many years. Specifically, this call asked for submissions that addressed one of three themes related to his program of research: (1) risk and protective factors in the development and maintenance of youth conduct problems, (2) family based preventive interventions for youth conduct problems, and (3) multicomponent preventive interventions for youth conduct problems. Reflecting Dr. McMahon’s scholarly productivity in both basic and applied empirical work, we encouraged submissions that presented novel, innovative, and cutting-edge research within the fields of prevention and intervention science and developmental psychopathology. What has resulted is a collection of 15 original articles that engage the science and legacy of Dr. McMahon’s work and highlight promising directions for future research in the field of youth conduct problems. Below, we provide a brief synopsis of each article that appears in the Special Issue, organized around the three themes related to Dr. McMahon’s program of research. We close this introductory article with reflections on future avenues for research on youth conduct problems, inspired by both his work and the scholarship contained within this Special Issue.

## Theme One: Risk and Protective Factors in the Development and Maintenance of Youth Conduct Problems

Students and mentees of Dr. McMahon will readily acknowledge that the main focus of his work, and the content of most scholarly conversations with him, falls within the area of youth externalizing psychopathology. He instilled in his students a deep understanding that in order to effectively prevent and intervene with youth conduct problems, one must first understand the genesis of that behavior—specifically, the risk and protective factors that lead to the development and maintenance of those behavior problems. This work has identified important parent (e.g., parental monitoring, harsh and inconsistent discipline), child (e.g., depression, callous-unemotional [CU] traits), and contextual (e.g., neighborhoods, schools) factors that are significantly related to specific manifestations of conduct problems (see for example, Goulter et al., 2020; Racz et al., 2019; Zheng & McMahon, 2022). While all of the articles within this Special Issue contain content relevant to understanding risk and protective factors for youth conduct problems, several of them have a direct focus on parenting and individual child/adolescent risk and protective factors.

Much of Dr. McMahon’s work regarding parenting illustrates the importance of (1) consistent and non-harsh discipline (e.g., Goulter et al., 2020; Pasalich et al., 2016; Zheng et al., 2017) and (2) developmentally appropriate parental monitoring (e.g., Dishion & McMahon, 1998; Pelham et al., 2024; Racz & McMahon, 2011) in order to effectively reduce child conduct problems and long-term antisocial behavior. While there is now an abundance of empirical evidence establishing negative parenting as a risk factor for youth conduct problems among parent–child dyads in Western samples, what is lacking are broader examinations considering intergenerational and international perspectives. Two papers in this Special Issue directly address this limitation. Specifically, Fosco and colleagues (2024) tested an intergenerational cascade model (G1: generation 1 → G2: generation 2 → G3: generation 3) and found that G1 family climate in adolescence was negatively associated with G2 antisocial behavior in early adulthood; in turn, this antisocial behavior was negatively associated with warm parenting and family cohesion and positively associated with family conflict and routines at G2 and G3. Additionally, Lansford and colleagues (2024) provide an international perspective on the developmental trajectories of a wide range of problem behaviors (i.e., conduct disorder, oppositional defiant disorder, alcohol/drug use, cigarette smoking, sexual activity), identifying four classes: late starters, alcohol experimenters, mid-adolescent starters, and pervasive risk takers. Positive parenting generally demonstrated protective effects from being in the pervasive risk taker class, while psychological control, parental monitoring/behavioral control, and autonomy granting posed risk for being in the pervasive risk takers class. The perspectives of these two papers are particularly important contributions to our understanding of how the associations between parenting and youth conduct problems stretch across generations and cultures.

Considering individual child risk factors for conduct problems, a significant portion of Dr. McMahon’s work has focused on CU traits. These traits are characterized as having a flat affect with restricted empathy and remorse and have been widely established as a strong correlate of youth conduct problems, leading to even greater antisocial behavior into adulthood (McMahon et al., 2010). In this Special Issue, Kemp and colleagues (2024) found that youth with elevated CU traits showed deficient responsiveness to negative emotional stimuli and that the association between poor emotion recognition and high levels of conduct problems was particularly strong when youth displayed high levels of CU traits. These findings add to the accumulating literature regarding the important role that CU traits play in manifestations of conduct problems and further suggest that addressing difficulties with emotion recognition, through emotion-coaching and other approaches, should be a key component of interventions for youth with elevated conduct problems and CU traits.

Dr. McMahon’s work on risk and protective factors for youth conduct problems also recognizes that a constellation of factors interact to increase risk for behavior problems (e.g., Racz et al., 2019; Zheng et al., 2017). In this Special Issue, Zheng and colleagues (2024) examined daily reciprocal associations between parenting behaviors (parental warmth, inconsistent discipline, and non-harsh discipline) and adolescent conduct problems and CU traits using a daily diary design. The authors found that both daily parental warmth and non-harsh discipline buffered against next-day adolescent CU traits, which was particularly strong for adolescents experiencing more generally positive parenting; the reciprocal direction of this effect was also supported for parental warmth. Additionally, Bellamy and colleagues (2024) identified associations between elevated levels of specific types of adolescent psychopathy (e.g., daring-impulsive traits) and lower parental monitoring behaviors and knowledge, which were weaker at high levels of adolescent-reported arousal. These findings suggest that understanding the interactions between various risk factors at multiple levels is essential for tailoring preventive interventions targeting youth conduct problems.

## Theme Two: Family-Based Preventive Interventions for Youth Conduct Problems

Some of Dr. McMahon’s earliest research advances occurred alongside his mentor Dr. Rex Forehand, as they developed, evaluated, and disseminated *Helping the Noncompliant Child* (HNC; Forehand & McMahon, 1981; McMahon & Forehand, 2003), a social learning-based parenting intervention designed to reduce coercive parent–child interactions, improve parenting skills, and decrease children’s problem behaviors. An adapted version of this program also provides supplemental emotion coaching to specifically target CU traits (Fainsilber Katz et al., 2025; McMahon et al., 2025). Various trials of HNC have demonstrated positive effects on outcomes 10–14 years post-treatment (McMahon et al., 2011). In this Special Issue, Highlander and colleagues (2024) found that parents with higher levels of financial strain prior to participation in HNC reported diminished gains in intensity and perception of child problem behavior at 6 months post-treatment, and a slower rate of change in perceptions of child problem behavior across treatment. These findings suggest that contextual risks may be particularly important moderators to consider when evaluating the effectiveness of family-based treatments for youth conduct problems.

In the broader literature, the Family Check-Up® (FCU) has also received extensive empirical support as a well-established, evidence-based treatment of youth conduct problems. FCU is a strengths-based, individualized intervention that aims to improve positive parenting skills and family management (Dishion & Kavanagh, 2003). In this Special Issue, Bennett and colleagues (2024) present promising findings from the first effectiveness evaluation of FCU for families of young children in Canada. Specifically, participants randomized to FCU reported lower child behavior problems at 12 months post-enrollment, relative to a comparison group self-referred to community mental health agencies. Additionally, Suarez and colleagues (2024) utilized data from a well-characterized, multisite randomized controlled trial (RCT) of FCU and found that inhibitory control in late childhood, as reported by parents, mediated the effect of the intervention as delivered in early childhood on reductions in adolescent-reported antisocial behavior. Importantly, this indirect effect was strongest for youth who resided in dangerous neighborhoods, affiliated with deviant peers, and reported low levels of parental knowledge and control, again highlighting important moderators of treatment effects.

Despite such promising results from RCTs and other effectiveness trials, it remains a significant challenge to engage families in treatment and ensure compliance with treatment sessions and goals, particularly for families experiencing significant contextual risks and whose children display clinically significant levels of conduct problems (Dishion et al., 2008). Reflecting on these difficulties, the future of family based treatments for youth conduct problems must innovate to effectively meet the needs of individual families. In this Special Issue, Stormshak and colleagues (2024) evaluated findings from two RCTs of the FCU Online (FCU-O), which is delivered to parents in brief format with supportive coaching that targets parenting skills, effortful control, and youth emotional problems. Findings from these analyses identified improvements in effortful control and emotional problems as important mediators of the effect of the FCU-O treatment on youth conduct problems at follow-up. Providing families with the opportunity to receive evidence-based treatments for youth conduct problems in a digital health format enhances accessibility and raises the potential for larger-scale implementation to reach youth and families who have difficulties accessing healthcare.

However, while recent technological advances have enabled clinicians to deliver intervention content digitally, what is lacking are rigorous empirical evaluations of the effectiveness of such approaches. To that end, in this Special Issue, Canário and colleagues (2024) present a meta-analysis of 27 RCTs of online parenting programs for improving children’s emotional and behavioral problems, revealing moderate effect sizes of these programs on reductions in child emotional and behavior problems and parents’ ineffective parenting behaviors and mental health problems. Several specific components of these online parenting programs were found to be particularly effective for targeting child emotional problems, including adoption of a learning theory perspective (e.g., positive reinforcement, non-violent discipline), a focus on parental self-care (e.g., stress reduction, problem solving), and utilization of “parents as therapists” approaches (e.g., parents actively teaching their children emotion regulation skills). Further reflecting on the importance of designing and implementing programs that meet the needs and preferences of families, Turner and Sanders (2024) highlight a systems-contextual approach to improve the reach of evidence-based parenting support. This theoretical piece proposes important considerations for future research studies that aim to effectively scale-up and disseminate established evidence-based interventions for families of youth with conduct problems.

## Theme Three: Multicomponent Preventive Interventions for Youth Conduct Problems

As one of the founding principal investigators for the Fast Track project, Dr. McMahon’s research legacy includes a consistent focus on using what is known about risk and protective factors for youth conduct problems to develop, test, and disseminate multicomponent prevention interventions targeting these problem behaviors. The Fast Track project is a longitudinal, multisite RCT aimed at preventing serious conduct problems (Conduct Problems Prevention Research Group, 2020). Fast Track comprises both high-risk and normative samples with half of the high-risk sample receiving a multicomponent intervention in grades 1–10, including parent management training (adapted primarily from HNC), child social and academic skills training, and a universal classroom program. Previous Fast Track trials have demonstrated positive intervention effects through age 25 (Dodge et al., 2015). In this Special Issue, McCabe and colleagues (2024) expanded these findings to age 31, showing that participation in the Fast Track intervention improved interpersonal skills in childhood, which in turn led to fewer externalizing, internalizing, and substance use problems; reduced criminality and number of sexual partners; and increased general health and full-time employment. These findings reveal important mechanistic pathways and suggest when and why the intervention was most effective.

A strength of multicomponent programs like the Fast Track project is their ability to address multiple risk factors simultaneously and across multiple settings to maximize generalizability of intervention gains. Interventions delivered in school settings can therefore be particularly effective, as they can target both interpersonal difficulties with peers as well as academic and other school-related challenges (e.g., poor school connectedness, negative teacher–student relationships). In this Special Issue, Heilman and colleagues (2025) identified important moderators of treatment effects from Coping Power, a school-based intervention for children aged 9–11 at risk for aggression. Specifically, as compared to group-delivered Coping Power, individually delivered Coping Power was more effective in reducing reactive aggression among youth with high levels of peer-rated acceptance but was less effective in reducing proactive aggression among those who underestimated their self-rated acceptance by their peers. Prinz and colleagues (2024) also present findings from a multicomponent preventive intervention delivered in schools to 1st and 2nd grade children residing in economically disadvantaged neighborhoods. Boys who participated in the intervention demonstrated better academic achievement in 3rd grade than those in the control group; however, these effects were not observed among girls or for behavioral or social adjustment outcomes, raising questions as to why these other outcomes were not affected by the intervention. Often, these questions can be answered with crucial “unpacking” studies to determine which aspects of a specific program are most effective (i.e., what are the “active ingredients?”) and how various program components work together to affect outcomes. Meta-analytic approaches are a promising technique to identify important moderators and mechanisms of change for these preventive interventions.

To that end, in this Special Issue, Craig and colleagues (2025) report on a meta-analysis and narrative review of the Stop Now and Plan (SNAP) program, a multicomponent preventive intervention targeting several hypothesized mechanisms underlying the development of conduct problems (e.g., emotion regulation, prosocial behaviors, parent–child relationships). Meta-analytic findings indicated a moderate effect size for SNAP program effects on conduct problems, and a narrative review presented promising findings regarding changes in the proposed mechanisms. Notably, the development and dissemination of SNAP has been facilitated by collaborative clinical–research partnerships and largely delivered in community mental health settings. Continued efforts to disseminate intervention trials, moving them out of academic settings and into communities, are key to Dr. McMahon’s research legacy of translating empirical evidence regarding youth conduct problems into effective prevention programming.

## Conclusion: The Future of Prevention Science and Youth Conduct Problems

The influence of Dr. McMahon’s body of work on the field of youth conduct problems is astutely and prominently reflected in the collection of empirical articles included in this Special Issue. Additionally, the scholarship contained within this Special Issue highlights many promising advances and future directions for the fields of developmental psychopathology and prevention science. These articles answer crucial questions about the development and treatment of youth conduct problems from longitudinal (Bennett et al., 2024; Fosco et al., 2024; Heilman et al., 2025; Highlander et al. 2024; Lansford et al., 2024; McCabe et al., 2024; Prinz et al., 2024; Stormshak et al., 2024; Suarez et al., 2024, Zheng et al., 2024), multi-informant (Bellamy et al., 2024; Kemp et al., 2024; Prinz et al., 2024; Suarez et al., 2024), intergenerational (Fosco et al., 2024), and international (Bennett et al., 2024; Lansford et al., 2024; Zheng et al., 2024) perspectives. They utilize large-scale RCTs to examine intervention effectiveness (Bennett et al., 2024; Heilman et al., 2025; McCabe et al., 2024; Suarez et al., 2024) and engage sophisticated modeling techniques to address important questions about how youth conduct problems manifest over time (e.g., Fosco et al., 2024; Lansford et al., 2024; Zheng et al., 2024). These articles encourage us to think about how to effectively intervene with families from diverse backgrounds (McCabe et al., 2024; Prinz et al., 2024; Suarez et al., 2024) and how to partner with community stakeholders to effectively disseminate evidence-based preventive interventions (Craig et al., 2025; Turner & Sanders, 2024). They also challenge us to develop innovative methods to deliver intervention content in ways that are easily accessible to families (Canário et al., 2024; Stormshak et al., 2024) in order to effectively “move the needle” and produce clinically significant and meaningful change in youth conduct problems. In this way, the articles within this Special Issue engage Dr. McMahon’s scholarly contributions, to use what we know from empirical investigations to drive actual change in the lives of children and families.

This Special Issue concludes with two commentaries from experts in the fields of prevention science and youth conduct problems (Greenberg, in press; Tolan, in press), as well as a reflection piece from Dr. McMahon. These articles synthesize the impressive scholarship contained within this Special Issue and present important and thought-provoking directions for future research. Reflecting on Dr. McMahon’s program of research and the articles within this Special Issue, we also see three important directions for the field.

First, advances in quantitative methodologies and statistical software capabilities allow researchers to investigate increasingly sophisticated and complex questions about youth conduct problems. Promising developments in longitudinal modeling, including daily diary and other microscale timing approaches, can investigate short-term fluctuations in conduct problems to better understand their dynamic development over time, beyond just group mean approaches and lag-1 longitudinal models (Zheng et al., 2024). Additional advances in dyadic data analysis, including actor–partner models, will also help address questions about how youth with conduct problems interact with important individuals in their lives (e.g., parents, siblings, peers, teachers; Li & Zheng, 2025). Further considering multimethod approaches (e.g., surveys, observational studies, laboratory designs) and different levels of analyses (e.g., biological perspectives) will also expand and improve our understanding and assessment of youth conduct problems. Capitalizing on these innovative research designs and statistical techniques will help push the science forward to address increasingly complex questions, better informing our understanding of how youth conduct problems develop over time and how to effectively intervene to interrupt their progression on multiple fronts.

Second, interventions for youth with conduct problems need to engage families more broadly, including utilizing telehealth and other digital health technologies, to effectively meet the needs of individual families and enhance accessibility to intervention options. This is particularly important for families that have difficulties accessing intervention (e.g., due to location or other contextual or historical risk factors that impede intervention engagement). Rigorous evaluations of digital health technologies and other innovations are needed to ensure that these intervention modalities are delivered ethically and with fidelity and efficacy. Additionally, implementation studies and dissemination trials need to consider any cultural adaptations that are necessary to address the specific needs of the communities in which these services are delivered. Large-scale RCTs also need to be conducted in diverse settings, including internationally and in Majority World countries, to move the science beyond academic settings and efficacy trials and ensure effective and adequate implementation of intervention programs across different populations globally. Attention to these issues is crucial to reduce any barriers to intervention options and ensure equitable access to intervention programming for those who need it the most.

Third, the field needs a much better understanding of which preventive interventions work, when, for whom, and why. Much of Dr. McMahon’s work, most notably through his role as a principal investigator on the Fast Track project, highlights the importance of developmentally informed, sustained, multicomponent prevention programming to impact clinically significant change in youth conduct problems. However, not all interventions work equally well for all families and for all youth with conduct problems. Studies that consider moderators and mediators of intervention effects are needed to better tailor interventions to the individual needs of youth and families and identify the “active ingredients” of intervention programs (Lochman et al., 2019; McMahon et al., 2021). Within the shifting landscape of mental health and managed care, being able to directly identify the most pressing needs of youth, and then apply the most effective aspects of an intervention program to target that need, will optimize intervention outcomes and impact meaningful change on youth conduct problems as they manifest in real-world settings. Inspired by Dr. McMahon’s research legacy, we see these as crucial directions for future investigations aimed at understanding how to effectively prevent and intervene on youth conduct problems, in order to interrupt the developmental progression of these behaviors over time.

## References

[CR1] Bellamy, N. A., Salekin, R. T., Racz, S. J., & De Los Reyes, A. (2024). A multi-dimensional, multi-informant examination of adolescent psychopathy and its links to parental monitoring: The moderating role of resting arousal. *Prevention Science*, 1–12. 10.1007/s11121-024-01753-z 10.1007/s11121-024-01753-z 39644384

[CR2] Bennett, T., Georgiades, K., Gonzalez, A., Janus, M., Lipman, E., Pires, P., Prime, H., Duku, E., Jambon, M., McLennan, J. D., Gross, J., & Making the Race Fair Study Team. (2024). Targeted child mental health prevention and parenting support within a Canadian context: A randomized controlled trial evaluating the US-developed Family Check-Up®. *Prevention Science*, 1–13. 10.1007/s11121-024-01741-3 10.1007/s11121-024-01764-w PMC12246013 39729256

[CR3] Canário, A. C., Pinto, R., Silva-Martins, M., Rienks, K., Akik, B. K., Stanke, K. M., David, O., Kızıltepe, R., Melendez-Torres, G. J., Thongseiratch, T., & Leijten, P. (2024). Online parenting programs for children’s behavioral and emotional problems: A network meta-analysis. *Prevention Science*, 1–18. 10.1007/s11121-024-01735-1 10.1007/s11121-024-01735-1 PMC12209006 39397230

[CR4] Conduct Problems Prevention Research Group. (2020). *The Fast Track program for children at risk: Preventing antisocial behavior*. Guilford Press.

[CR5] Craig, S. G., Frankiewicz, K., Stearns, N. R., Girard-Lapointe, J., Cortese, A., Vogel, N., & Pepler, D. J. (2025). What do we know about the Stop Now and Plan Program? A systematic review and meta-analysis of an early invention for children and youth with conduct problems. *Prevention Science*, 1–23. 10.1007/s11121-025-01788-w 10.1007/s11121-025-01788-w 39951237

[CR6] Dishion, T. J., & Kavanagh, K. (2003). *Intervening in adolescent problem behavior: A family-centered approach*. Guilford Press.

[CR7] Dishion, T. J., & McMahon, R. J. (1998). Parental monitoring and the prevention of child and adolescent problem behavior: A conceptual and empirical formulation. *Clinical Child and Family Psychology Review,**1*(1), 61–75. 10.1037/e495502006-010 11324078 10.1023/a:1021800432380

[CR8] Dishion, T. J., Shaw, D., Connell, A., Gardner, F., Weaver, C., & Wilson, M. (2008). The family check-up with high-risk indigent families: Preventing problem behavior by increasing parents’ positive behavior support in early childhood. *Child Development,**79*(5), 1395–1414. 10.1111/j.1467-8624.2008.01195.x 18826532 10.1111/j.1467-8624.2008.01195.x PMC2683384

[CR9] Dodge, K. A., Bierman, K. L., Coie, J. D., Greenberg, M. T., Lochman, J. E., McMahon, R. J., & Pinderhughes, E. E. (2015). Impact of early intervention on psychopathology, crime, and well-being at age 25. *American Journal of Psychiatry,**172*(1), 59–70. 10.1176/appi.ajp.2014.13060786 25219348 10.1176/appi.ajp.2014.13060786 PMC4485380

[CR10] Fainsilber Katz, L., Kerns, S. E. U, Gurtovenko, K., Pasalich, D., Goulter, N., Dorsey, S., Pullmann, M. D., Dawson, A., Galtieri, L. R., & McMahon, R. J. (2025). *Integrating parent management and emotion coaching for young children with callous-unemotional traits*. [Manuscript submitted for publication]. 10.1016/j.appdev.2025.101808 PMC12345356 40814353

[CR11] Forehand, R. L., & McMahon, R. J. (1981). *Helping the noncompliant child: A clinician’s guide to parent training*. Guilford Press.

[CR12] Fosco, G. M., Van Ryzin, M. J., Feinberg, M. E., & Lee, H. (2024). Cascading effects of the family context in adolescence: Implications for young adult antisocial behavior and intergenerational transmission of risk. *Prevention Science*, 1–12. 10.1007/s11121-024-01727-1 10.1007/s11121-024-01727-1 39422818

[CR13] Frick, P. J. (2012). Developmental pathways to conduct disorders: Implications for future directions in research, assessment, and treatment. *Journal of Clinical Child & Adolescent Psychology,**41*(3), 378–389. 10.1080/15374416.2012.664815 22475202 10.1080/15374416.2012.664815

[CR14] Goulter, N., McMahon, R. J., Pasalich, D. S., & Dodge, K. A. (2020). Indirect effects of early parenting on adult antisocial outcomes via adolescent conduct disorder symptoms and callous-unemotional traits. *Journal of Clinical Child & Adolescent Psychology*, *49*(6), 930–942. 10.1080/15374416.2019.1613999 10.1080/15374416.2019.1613999 PMC6893105 31166154

[CR42] Greenberg, M. (in press). Honoring the work of Robert McMahon: A commentary on this special issue. *Prevention Science*. 10.1007/s11121-025-01807-w PMC12209007 40257701

[CR15] Heilman, M. E., Lochman, J. E., Laird, R. D., McDonald, K. L., Barth, J. M., Powell, N. P., Boxmeyer, C. L., & White, B. A. (2025). Can peer acceptance and perceptual accuracy impact the effectiveness of two formats of a preventative intervention on functional subtypes of aggression in youth? *Prevention Science*, 1–12. 10.1007/s11121-025-01767-1 10.1007/s11121-025-01767-1 39806163

[CR16] Highlander, A., Parent, J., & Jones, D. J. (2024). Helping the noncompliant child and child behavior outcomes: An exploratory examination of financial strain. *Prevention Science*, 1–13. 10.1007/s11121-024-01749-9 10.1007/s11121-024-01749-9 PMC12059154 39514027

[CR17] Kemp, E. C., Clark, J. E., Matlasz, T. M., & Frick, P. J. (2024). Associations between callous-unemotional (CU) traits and emotion recognition abilities in school children: The influence of conduct problems and age. *Prevention Science*, 1–13. 10.1007/s11121-024-01746-y 10.1007/s11121-024-01746-y 39508964

[CR18] Kimonis, E. R., Frick, P. J., & McMahon, R. J. (2014). Conduct and oppositional defiant disorders. In E. J. Mash & R. A. Barkley (Eds.), *Child psychopathology* (3^rd^ ed., pp. 145–179). Guilford Press.

[CR19] Lansford, J. E., Godwin, J., Rothenberg, W. A., Alampay, L. P., Al-Hassan, S. M., Bacchini, D., Bornstein, M. H., Chang, L., Deater-Deckard, K., Di Giunta, L., Dodge, K. A., Gurdal, S., Junla, D., Oburu, P., Pastorelli, C., Skinner, A. T., Sorbring, E., Steinberg, L., Tirado, L. M. U., & Yotanyamaneewong, S. (2024). Parenting risk and protective factors in the development of conduct problems in seven countries. *Prevention Science*, 1–13. 10.1007/s11121-024-01743-1 10.1007/s11121-024-01743-1 PMC12276935 39508965

[CR20] Li, K., & Zheng, Y. (2025). Convergence and divergence in adolescent- and parent-reported daily parental positive reinforcement: Dynamic links with adolescent emotional and behavioral problems. *Developmental Psychology*, 1–15. 10.1037/dev0001947 10.1037/dev0001947 40014527

[CR21] Lochman, J. E., Boxmeyer, C. L., Kassing, F. L., Powell, N. P., & Stromeyer, S. L. (2019). Cognitive-behavioral interventions for youth at-risk for conduct problems: Future directions. *Journal of Clinical Child & Adolescent Psychology,**48*(5), 799–810. 10.1080/15374416.2019.1567349 30892949 10.1080/15374416.2019.1567349 PMC6710135

[CR22] McCabe, G., Godwin, J. W., Rothenberg, W. A., Goulter, N., Lansford, J. E., & Conduct Problems Prevention Research Group. (2024). Fast Track intervention effects and mechanisms of action through established adulthood. *Prevention Science*, 1–14. 10.1007/s11121-024-01736-0 10.1007/s11121-024-01736-0 PMC12034041 39392546

[CR23] McMahon, R. J., & Forehand, R. L. (2003). *Helping the noncompliant child: Family-based treatment for oppositional behavior* (2^nd^ ed.). Guilford Press.

[CR24] McMahon, R. J., & Frick, P. J. (2005). Evidence-based assessment of conduct problems in children and adolescents. *Journal of Clinical Child & Adolescent Psychology,**34*(3), 477–505. 10.1207/s15374424jccp3403_6 16026215 10.1207/s15374424jccp3403_6

[CR25] McMahon, R. J., Goulter, N., & Frick, P. J. (2021). Moderators of psychosocial intervention response for children and adolescents with conduct problems. *Journal of Clinical Child & Adolescent Psychology,**50*(4), 525–533. 10.1080/15374416.2021.1894566 33787407 10.1080/15374416.2021.1894566

[CR26] McMahon, R. J., Kerns, S. E. U, Gurtovenko, K., Goulter, N., Pasalich, D. S., Dawson, A., Galtieri, L. R., Dorsey, S., Pullmann, M. D., & Fainsilber Katz, L. (2025). *Pilot RCT integrating PMT and emotion coaching for children with callous-unemotional traits*. [Manuscript submitted for publication]. 10.1016/j.appdev.2025.101808 PMC12345356 40814353

[CR27] McMahon, R. J., & Kotler, J. S. (2004). Treatment of conduct problems in children and adolescents. In P. M. Barrett & T. H. Ollendick (Eds.), *Handbook of interventions that work with children and adolescents: Prevention and treatment* (pp. 395–425). Wiley.

[CR28] McMahon, R. J., Long, N., & Forehand, R. L. (2011). Parent training for the treatment of oppositional behavior in young children: Helping the noncompliant child. In R. Murrihy, A. Kidman, & T. Ollendick (Eds.), *Clinical handbook of assessing and treating conduct problems in youth* (pp. 163–191). Springer.

[CR29] McMahon, R. J., Witkiewitz, K., Kotler, J. S., & Conduct Problems Prevention Research Group. (2010). Predictive validity of callous–unemotional traits measured in early adolescence with respect to multiple antisocial outcomes. *Journal of Abnormal Psychology,**119*(4), 752–763. 10.1037/a0020796 20939651 10.1037/a0020796 PMC3760169

[CR30] Pasalich, D. S., Witkiewitz, K., McMahon, R. J., Pinderhughes, E. E., & Conduct Problems Prevention Research Group. (2016). Indirect effects of the fast track intervention on conduct disorder symptoms and callous-unemotional traits: Distinct pathways involving discipline and warmth. *Journal of Abnormal Child Psychology,**44*(3), 587–597. 10.1007/s10802-015-0059-y 26242993 10.1007/s10802-015-0059-y PMC4961049

[CR31] Pelham, W. E., III., Racz, S. J., Davis, I. S., Aks, I. R., Patel, H., McMahon, R. J., Thornburg, M. A., Huang, Y.-T.W., Schulze, E. M., Gonzalez, O., Tapert, S. F., & Brown, S. A. (2024). What is parental monitoring? *Clinical Child and Family Psychology Review,**27*, 576–601. 10.1007/s10567-024-00490-7 38869680 10.1007/s10567-024-00490-7 PMC11801412

[CR32] Prinz, R. J., Smith, E. P., & Tennie, B. (2024). A multicomponent preventive intervention in the early elementary years: A look at academic and social adjustment outcomes. *Prevention Science*, 1–11. 10.1007/s11121-024-01748-w 10.1007/s11121-024-01748-w PMC12209043 39523255

[CR33] Racz, S. J., & McMahon, R. J. (2011). The relationship between parental knowledge and monitoring and child and adolescent conduct problems: A 10-year update. *Clinical Child and Family Psychology Review*, *14*(4), 377–398. 10.1007/s10567-011-0099-y 10.1007/s10567-011-0099-y 22086648

[CR34] Racz, S. J., McMahon, R. J., King, K. M., Pinderhughes, E. E., & Bendezú, J. J. (2019). Kindergarten antecedents of the developmental course of active and passive parental monitoring strategies during middle childhood and adolescence. *Development and Psychopathology,**31*(5), 1675–1694. 10.1017/S0954579419000993 31718735 10.1017/S0954579419000993 PMC7055711

[CR35] Stormshak, E., Connell, A., Mauricio, A. M., McLaughlin, M., & Caruthers, A. (2024). Digital health delivery of parenting skills to improve conduct problems in middle school youth across two distinct randomized trials. *Prevention Science*, 1–10. 10.1007/s11121-024-01750-2 10.1007/s11121-024-01750-2 PMC12208997 39556238

[CR36] Suarez, G. L., Shaw, D. S., Wilson, M. N., Lemery-Chalfant, K., & Hyde, L. W. (2024). Inhibitory control in late childhood as a predictor of antisocial behavior in adolescence and the role of social context. *Prevention Science*, 1–14. 10.1007/s11121-024-01754-y 10.1007/s11121-024-01754-y PMC12086261 39562476

[CR41] Tolan, P. H. (in press). The multiple avenues of contribution of Robert McMahon to prevention science. *Prevention Science.* 10.1007/s11121-025-01809-8 PMC12208952 40259177

[CR37] Turner, K. M., & Sanders, M. R. (2024). Finding solutions to scaling parenting programs that work: A systems-contextual approach. *Prevention Science*, 1–11. 10.1007/s11121-024-01755-x 10.1007/s11121-024-01755-x 39592561

[CR38] Zheng, Y., Li, K., Zheng, H., & Pasalich, D. S. (2024). Daily associations between parental warmth and discipline and adolescent conduct problems and callous-unemotional traits. *Prevention Science*, 1–11. 10.1007/s11121-024-01740-4 10.1007/s11121-024-01740-4 39377986

[CR39] Zheng, Y., & McMahon, R. J. (2022). Lability in parental warmth in childhood: Antecedents and early adolescent outcomes. *Journal of Clinical Child & Adolescent Psychology,**51*(5), 610–622. 10.1080/15374416.2019.1678166 31670982 10.1080/15374416.2019.1678166 PMC7190436

[CR40] Zheng, Y., Pasalich, D. S., Oberth, C., McMahon, R. J., & Pinderhughes, E. E. (2017). Capturing parenting as a multidimensional and dynamic construct with a person-oriented approach. *Prevention Science,**18*(3), 281–291. 10.1007/s11121-016-0665-0 27179538 10.1007/s11121-016-0665-0 PMC6141018

